# Correlation of Lung Texture Analysis by Computer-Aided Lung Informatics for Pathology Evaluation and Rating (CALIPER) With High-Resolution Computed Tomography and Lung Function in Fibrosing Interstitial Lung Diseases

**DOI:** 10.7759/cureus.108104

**Published:** 2026-05-01

**Authors:** Pravitha S, John Sonia Kallarakal, Venugopal Panikar, Venugopal K.P., Sajitha M

**Affiliations:** 1 Pulmonary Medicine, Government Medical College, Kottayam, Kottayam, IND; 2 Pulmonology, Government Medical College, Kottayam, Kottayam, IND

**Keywords:** 6mwt, artificial intelligence, caliper, fibrosing ild, forced vital capacity, lung texture analysis, quantitative ct scoring, warrick hrct scoring

## Abstract

Background

Progressive pulmonary fibrosis (PPF) is characterized by worsening symptoms, physiological decline (forced vital capacity (FVC) or diffusing capacity for carbon monoxide), and/or radiological progression within a year. It can develop in patients with fibrosing interstitial lung diseases (ILDs), including idiopathic pulmonary fibrosis (IPF) and other non-IPF ILDs. Confounding variables and intra-individual variability limit the utility of pulmonary function tests. Interobserver variability affects the prognostic value of assessments based on high-resolution CT (HRCT), such as the semi-quantitative Warrick score. This study aimed to evaluate whether quantitative lung texture analysis (LTA) using the Computer-Aided Lung Informatics for Pathology Evaluation and Rating (CALIPER) software (Mayo Clinic, Rochester, MN) provides an accurate assessment of fibrosis and corresponds with semi-quantitative HRCT scores and functional measures in fibrosing ILDs.

Aim

To study the correlation between LTA by CALIPER with chest HRCT findings and lung function in fibrosing ILDs. A secondary aim was to determine the utility of LTA by CALIPER in identifying progression in fibrosing ILDs.

Methods

A total of 57 consecutive patients with fibrosing ILD were included in this hospital-based prospective study conducted in the Department of Pulmonary Medicine, Government Medical College, Kottayam, over a period of 12 months. Patients underwent detailed evaluation, including clinical assessment, semi-quantitative Warrick scoring of HRCT of the chest, FVC by spirometry, six-minute walk test (6MWT), and quantitative LTA obtained from CALIPER at baseline and at six-month follow-up to assess progression. Progression was assessed using changes in HRCT scores, pulmonary function, exercise desaturation, and predefined percentage cut-offs (2%, 5%, 10%, and 20% changes in normal lung percentage by LTA). Data were analyzed using SPSS version 25.0 (IBM Corp., Armonk, NY) with the Spearman correlation test, and associations between CALIPER measures, pulmonary function tests, and HRCT findings were evaluated (p < 0.05 was considered significant).

Results

Out of 57 patients, 38 (66.7%) were females, with a mean age of 57.84 ± 11.1 years. The most common diagnosis was connective tissue disease-related ILD, followed by idiopathic nonspecific interstitial pneumonia (NSIP). Baseline LTA ground-glass (GG) scores showed a moderate to strong positive correlation with the Warrick alveolitis index (r = 0.675, p < 0.001) and at six months (r = 0.700, p < 0.001). Similarly, LTA fibrosis scores demonstrated significant moderate positive correlations with the Warrick fibrosis index at baseline (r = 0.656, p < 0.001) and at six months (r = 0.631, p < 0.001). Among functional parameters, the strongest correlation was observed between LTA fibrosis score at six months and 6MWT desaturation (r = 0.731, p < 0.001). A 5% reduction in normal lung volume on CALIPER showed diagnostic accuracy of 68.42%, with sensitivity of 51.72% and specificity of 85.71% for predicting disease progression.

Conclusions

Quantitative LTA using CALIPER demonstrates a strong correlation with semi-quantitative HRCT scoring and a significant correlation with functional indices. CALIPER may serve as a useful adjunct in the routine monitoring of fibrosing ILDs and aid in the early identification of patients at risk for PPF at the earliest.

## Introduction

Fibrosing interstitial lung diseases: Clinical spectrum, pathobiology, and disease burden

Fibrosing interstitial lung diseases (ILDs) represent a diverse group of chronic disorders characterized by varying degrees of inflammation and fibrosis involving the pulmonary interstitium. Although heterogeneous in etiology, these conditions share a common final pathway of progressive scarring of the lung parenchyma, ultimately leading to respiratory impairment. Idiopathic pulmonary fibrosis (IPF) is the most extensively studied form and is considered the prototype of progressive fibrosing ILD [1].

Initially, the pathogenesis of ILD was unclear. However, our current understanding of its pathogenesis has evolved significantly. Fibrosing ILDs, particularly IPF, are now understood to arise not only from chronic inflammation but predominantly from repetitive alveolar epithelial injury, followed by aberrant wound-healing responses. This leads to fibroblast proliferation, myofibroblast activation, and excessive deposition of extracellular matrix proteins, progressively distorting the normal lung architecture [2]. The consequence is stiffening of lung tissue, impaired oxygen diffusion, and gradual respiratory failure.

The burden of ILD in India is increasingly recognized. Data from the ILD India Registry, one of the largest prospective registries from the country, revealed that hypersensitivity pneumonitis (HP) is the most common ILD subtype in India, followed by connective tissue disease (CTD)-associated ILD (CTD-ILD) and IPF [3]. This distribution differs from Western cohorts, where IPF predominates, highlighting geographical and environmental influences on disease patterns. Importantly, among patients with systemic sclerosis and rheumatoid arthritis, morbidity and mortality are largely attributed to CTD-associated ILDs [4].

Definition of progressive pulmonary fibrosis

In a patient diagnosed with ILD of either identified or unidentified cause, excluding IPF, and who demonstrates radiologic features consistent with pulmonary fibrosis, progressive pulmonary fibrosis (PPF) is defined by the presence of at least two out of three specified criteria within the previous year, provided no other cause explains the changes [5]. These criteria include worsening respiratory symptoms over time, objective physiological evidence of disease progression, and radiological evidence of progression. Physiological progression is defined by an absolute decline in forced vital capacity (FVC) of more than 5% predicted within one year of follow-up or an absolute decline in diffusing capacity of the lungs for carbon monoxide (DLCO), corrected for hemoglobin, of more than 10% predicted within the same period. Radiological progression is characterized by features such as an increased extent or severity of traction bronchiectasis or bronchiolectasis, the appearance of new ground-glass opacities in association with traction bronchiectasis, new fine reticulation, an increase in the extent or coarseness of reticular abnormalities, the development or progression of honeycombing, or evidence of increased lobar volume loss.

The utility of longitudinal physiological indices (decline in FVC and/or DLCO over six to 12 months) for assessing progression in fibrosing ILDs is still significantly limited by substantial intra-individual variability, perhaps with confounding by coexisting conditions such as emphysema or pulmonary hypertension [6].

Progressive fibrotic phenotypes, irrespective of underlying cause, are associated with declining lung function and poor survival [7]. Given the significant disease burden and impact on quality of life, accurate assessment of disease severity and progression is critical in pulmonology practice.

Role of high-resolution computed tomography in the evaluation of fibrosing ILDs

High-resolution computed tomography (HRCT) has become the cornerstone of ILD diagnosis. Its ability to provide a detailed visualization of lung parenchyma allows clinicians to identify specific radiologic patterns that guide diagnosis and management. Current international guidelines recognize that in appropriate clinical settings, a typical usual interstitial pneumonia (UIP) pattern on HRCT is sufficient to establish a diagnosis of IPF without the need for surgical lung biopsy [1].

HRCT features of fibrosing ILDs include reticulation, traction bronchiectasis, architectural distortion, and honeycombing. Among these, honeycombing has consistently been associated with more advanced disease and worse prognosis [8]. The visual extent of fibrosis on HRCT has also been shown to correlate with survival outcomes.

Indian studies have further described the radiologic spectrum of ILD in the local population. Chronic HP, which is common in India, often demonstrates fibrotic changes with upper-lobe predominance and features of air-trapping [9]. However, the visual interpretation of HRCT has limitations. Interobserver variability remains significant, particularly in differentiating early honeycombing from traction bronchiolectasis and in distinguishing fine reticulation from ground-glass opacities [10].

Therefore, while HRCT remains indispensable in the evaluation of fibrosing ILDs, its semi-quantitative and observer-dependent nature underscores the need for more objective and reproducible tools.

Quantitative CT and lung texture analysis in fibrosing interstitial lung diseases

Advances in imaging analysis have led to the development of quantitative CT (QCT) techniques that allow objective assessment of lung parenchymal abnormalities. Unlike visual scoring, these methods analyze volumetric HRCT data using computational algorithms to quantify the extent of different lung patterns.

Although several platforms are currently under investigation, the most widely studied automated analysis platform in ILD is CALIPER (Computer-Aided Lung Informatics for Pathology Evaluation and Rating; Mayo Clinic, Rochester, MN). It segments lung parenchyma and classifies tissue into categories such as normal lung, ground-glass opacity, reticulation, honeycombing, and vessel-related structures (VRS).

In systemic sclerosis-associated ILD, QCT assessment has been shown to be sensitive in detecting longitudinal disease progression and provides complementary information to visual scoring [11]. Although data from India regarding CALIPER application are limited, the increasing availability of high-quality volumetric HRCT scans in tertiary centers presents an opportunity for its integration into routine evaluation.

Automated texture analysis offers improved reproducibility and may reduce observer variability, making it a promising adjunct in ILD assessment.

Pulmonary function impairment and its relationship with structural lung abnormalities

Pulmonary function tests (PFTs) remain fundamental to the assessment of fibrosing ILDs. A restrictive pattern in spirometry with decreased FVC and total lung capacity (TLC), along with worsening of six-minute walk test (6MWT) parameters, is characteristic.

FVC decline is widely used as a marker of disease progression and was the primary endpoint in major antifibrotic trials, including the INPULSIS trial evaluating nintedanib [12]. However, PFTs provide only a global measure of lung function and may not fully reflect regional disease heterogeneity.

Prior studies have demonstrated that a greater fibrotic extent on CT correlates with lower FVC [13]. Moreover, automated CT quantification has demonstrated stronger correlations with functional decline compared to visual scoring methods [14,15]. In clinical practice, radiologic progression may at times precede measurable functional deterioration, particularly in early disease.

Therefore, the present study aims to evaluate whether computer-aided texture analysis of HRCT chest images can provide a precise and reproducible assessment of fibrosing ILD and whether these quantitative findings correlate with visual HRCT assessment and pulmonary function parameters. The secondary objective is to investigate whether longitudinal changes in parenchymal features, as quantified by CALIPER software, correlate with functional parameters for the prediction of progressive phenotype in fibrosing ILDs.

Objectives

The primary objectives of the present study are to evaluate the correlation between CALIPER-based lung texture analysis (LTA) and visual HRCT assessment in fibrosing ILDs and to determine the relationship between CALIPER-derived QCT parameters and pulmonary function parameters in affected patients. The secondary objective is to assess the utility of LTA using CALIPER in identifying disease progression in fibrosing ILDs.

## Materials and methods

Study design, period, and setting

This was a hospital-based prospective study conducted over a period of 12 months from the date of Institutional Review Board (IRB) approval. The study was carried out in the Department of Pulmonary Medicine at Government Medical College, Kottayam, India.

Sampling and sample size

The sample size for the study was calculated using a standard formula for correlation studies, incorporating a Z value corresponding to a two-sided alpha error of 0.05 (Zα = 1.96) and a beta error of 0.20 (Zβ = 0.84). A correlation coefficient (r) of 0.38 was assumed based on a prior study by Raghunath et al. [11]. Using these parameters, the calculated sample size was 52. To account for a potential attrition rate of approximately 10%, the final sample size was increased to 57 patients. The inclusion and exclusion criteria are shown in Table 1.

**Table 1:** Inclusion and exclusion criteria of the study.

Inclusion criteria	Exclusion criteria
Consecutive patients diagnosed with fibrosing interstitial lung disease during the study period	Active infection
Age >18 years	Unable to perform the six-minute walk test or pulmonary function test due to poor functional status
	On antifibrotic therapy
	Diagnosed with idiopathic pulmonary fibrosis

Study population

All consecutive patients diagnosed with fibrosing ILD during the study period at the Department of Pulmonary Medicine, Government Medical College, Kottayam, were included in the study.

Study tools

Data collection and evaluation were carried out using a structured case proforma, HRCT of the chest, spirometry for pulmonary function assessment, and quantitative LTA using CALIPER software.

Study procedure

All consecutive patients diagnosed with fibrosing ILDs during the study period at the Department of Pulmonary Medicine, Government Medical College, Kottayam, were included in the study. Demographic data collected included age, sex, and smoking history (expressed as pack-years).

HRCT of the chest was evaluated using the Warrick score, a semi-quantitative system based on the type and extent of the parenchymal abnormalities. The total Warrick score (range = 0-30) is calculated by combining severity and extent scores for specific lesions (e.g., ground-glass opacities, irregular pleural margins, septal/subpleural lines, honeycombing, and subpleural cysts), with higher scores indicating a greater radiological involvement. The alveolitis index is determined by the presence and extent of ground-glass opacities (range = 0-4). The fibrosis index is assessed based on irregularities in pleural margins, septal or subpleural lines, honeycombing appearance, and subpleural cysts (range = 0-26).

Quantitative LTA was performed using CALIPER software, a validated computational tool for near-real-time characterization and quantification of lung parenchymal patterns on CT scans. The analysis was conducted on a set of images utilizing a standard reconstruction algorithm.

All patients underwent a comprehensive evaluation to identify underlying autoimmune or connective tissue diseases. PFTs, including spirometry, were performed within 30 days of the selected scans, in accordance with the standards established by the American Thoracic Society/European Respiratory Society guidelines. FVC and forced expiratory volume in one second (FEV₁) were expressed as percentages of predicted values using appropriate reference equations. The 6MWT was also conducted, with distance, desaturation, and other parameters recorded.

Follow-up assessments were done at six months and included assessing worsening dyspnea, a decline in FVC in PFT, desaturation during the 6MWT, and radiological progression on HRCT through visual scoring (Warrick’s) and quantitative LTA.

Disease progression on the Warrick HRCT scoring method was defined as an increase in the total score or a deterioration in individual scores for specific abnormalities when compared to the baseline. This progression may be evident as an increase in the extent or severity of fibrosis, the emergence of new abnormalities, or the enlargement of existing lesions.

For LTA using CALIPER, four thresholds were examined (2%, 5%, 10%, and 20%) to determine a change from the baseline. Disease progression was defined as a decrease in the percentage of normal lung pattern exceeding the threshold cut off, improvement as an increase exceeding the cut off, and stability if changes were below the thresholds.

Data management and analysis

Data were entered in Microsoft Excel (Microsoft Corporation, Redmond, WA), and statistical analysis was performed using SPSS version 25.0 (IBM Corp., Armonk, NY). Descriptive statistics were used to summarize the data. Categorical variables such as gender, occupation, dyspnea grade, associated CTD, comorbidities, and serological factors were expressed as frequency and percentage. Continuous variables such as age, dyspnea score, FVC, 6MWT distance, 6MWT desaturation, Warrick scores, and LTA scores were expressed as mean and standard deviation.

The association between LTA parameters (ground glass score, fibrosis score, and normal lung percentage) and clinical/radiological variables (Warrick score, FVC, 6MWT distance, and 6MWT desaturation) was analyzed using the Spearman correlation test. Correlation coefficients (r) were calculated, and their statistical significance was determined. Correlation coefficients were interpreted based on standard thresholds, with values between 0.90 and 1.00 indicating very high correlation, 0.70 to 0.90 indicating high correlation, 0.50 to 0.70 indicating moderate correlation, 0.30 to 0.50 indicating low correlation, and values below 0.30 indicating negligible correlation, with corresponding negative values representing inverse relationships [16].

The diagnostic performance of various CALIPER thresholds (2%, 5%, 10%, and 20%) for detecting PPF was evaluated by calculating sensitivity, specificity, positive predictive value (PPV), negative predictive value (NPV), and overall accuracy in comparison with the reference standard for PPF.

Receiver operating characteristic (ROC) curve analysis was performed to assess the discriminatory ability of delta lung percentage (change in percentage normal lung pattern) in predicting PPF. The area under the curve (AUC), standard error, 95% confidence interval, and optimal cut-off value were determined. A p-value of less than 0.05 was considered statistically significant.

## Results

Study population

A total of 57 patients with fibrosing ILDs were included in the present study and followed for six months.

Age distribution

The mean age of the study population was 57.84 ± 11.1 years (95% confidence interval (CI): 54.9-60.8 years). The youngest patient was 32 years old, while the oldest was 77 years old. The age-wise distribution of the study population is shown in Table 2.

**Table 2:** Age-wise distribution of the study population.

Age group	Number (n)	Percentage (%)
<40 years	5	8.80%
40-60 years	28	49.10%
>60 years	24	42.10%

Gender distribution

**Figure 1:**
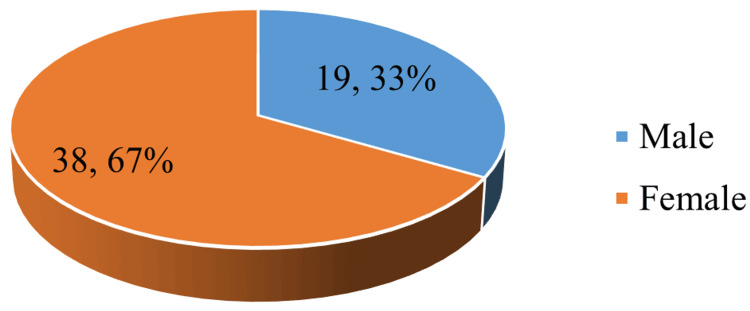
Gender distribution.

Among the 57 patients, 38 (66.67%) were female, and 19 (33.33%) were male, demonstrating a female predominance in this study population. Figure 1 depicts the gender distribution of the study subjects.

Occupational distribution

**Figure 2:**
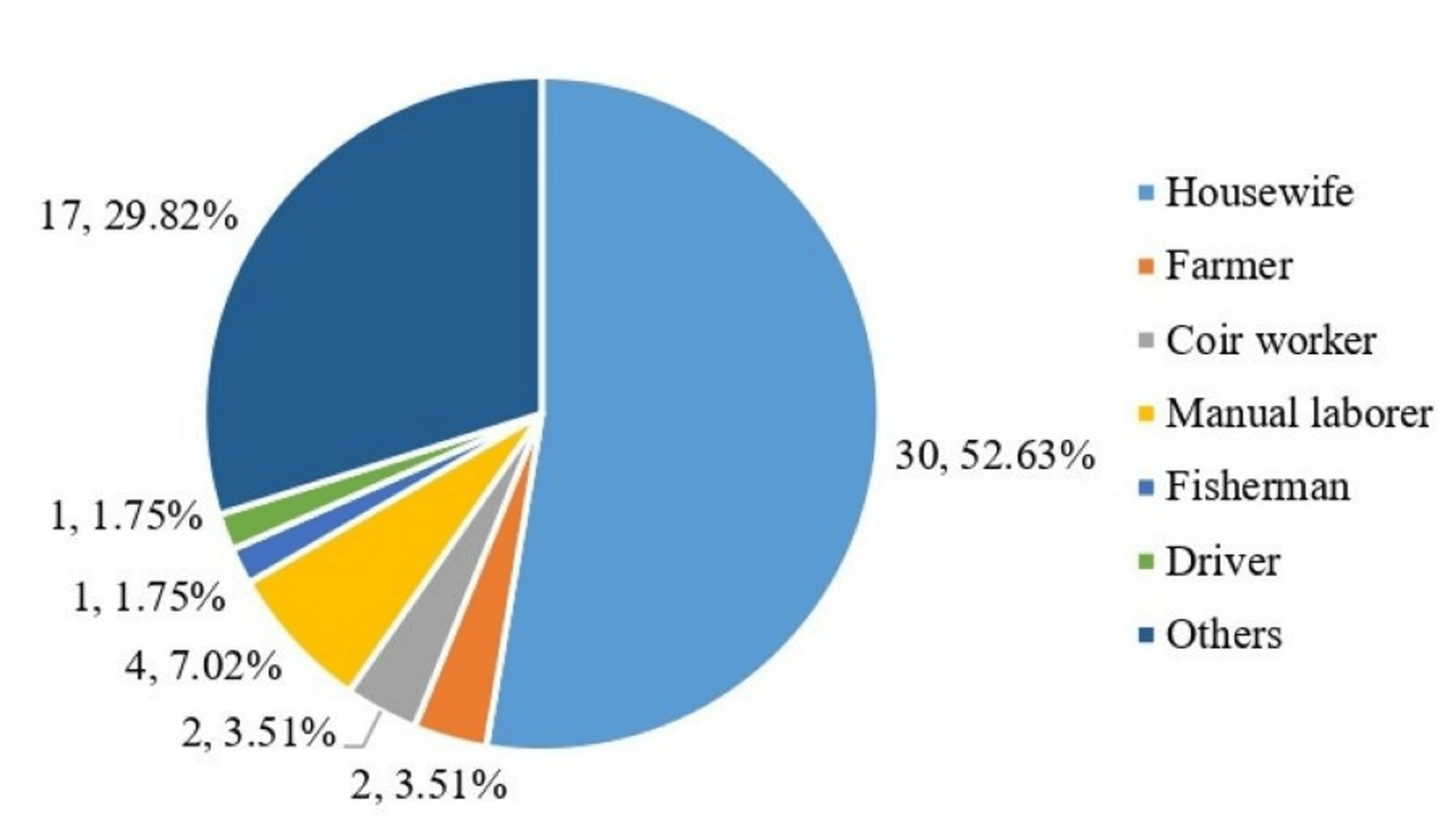
Occupational distribution.

The majority of patients were housewives (30, 52.6%). Other occupations included farmers (2, 3.5%), coir workers (2, 3.5%), manual laborers (4, 7.0%), fishermen (1, 1.8%), drivers (1, 1.8%), and others (17, 29.8%). The occupational distribution is shown in Figure 2.

Clinical profile

The grading of dyspnea at baseline and at six months is shown in Table 3. At baseline, 78.9% (n = 45) of patients had grade 1 dyspnea, 19.3% (n = 11) had grade 2, 1.8% (n = 1) had grade 3, and none had grade 4. At the six-month follow-up, 21.1% (n = 12) remained in grade 1, 36.8% (n = 21) were in grade 2, 38.6% (n = 22) in grade 3, and 3.5% (n = 2) were in grade 4.

**Table 3:** Dyspnea grading.

Condition	Status	n	%
Dyspnea (baseline)	Grade 1	45	78.95%
	Grade 2	11	19.30%
	Grade 3	1	1.75%
	Grade 4	0	0.00%
Dyspnea (six months)	Grade 1	12	21.05%
	Grade 2	21	36.84%
	Grade 3	22	38.60%
	Grade 4	2	3.51%

The associated CTDs are shown in Table 4. Eighteen patients (31.58%) had no associated CTD. Among the remaining patients, rheumatoid arthritis was present in 12 patients (21.1%), anti-synthetase syndrome in six (10.5%), systemic sclerosis in three (5.3%), Sjogren’s syndrome in one (1.8%), anti-MDA5 positivity in one (1.8%), dermatomyositis in one (1.8%), and idiopathic nonspecific interstitial pneumonia (NSIP) in 15 (26.3%).

**Table 4:** Associated connective tissue diseases. Anti-MDA5: anti-melanoma differentiation associated protein 5 antibody; NSIP: non-specific interstitial pneumonia.

Associated connective tissue disease	Number (n)	Percentage (%)
Anti-synthetase	6	10.53%
Rheumatoid arthritis	12	21.05%
Systemic sclerosis	3	5.26%
Sjogren’s	1	1.75%
Anti-MDA5	1	1.75%
Dermatomyositis	1	1.75%
Idiopathic NSIP	15	26.32%
Nil	18	31.58%

**Figure 3:**
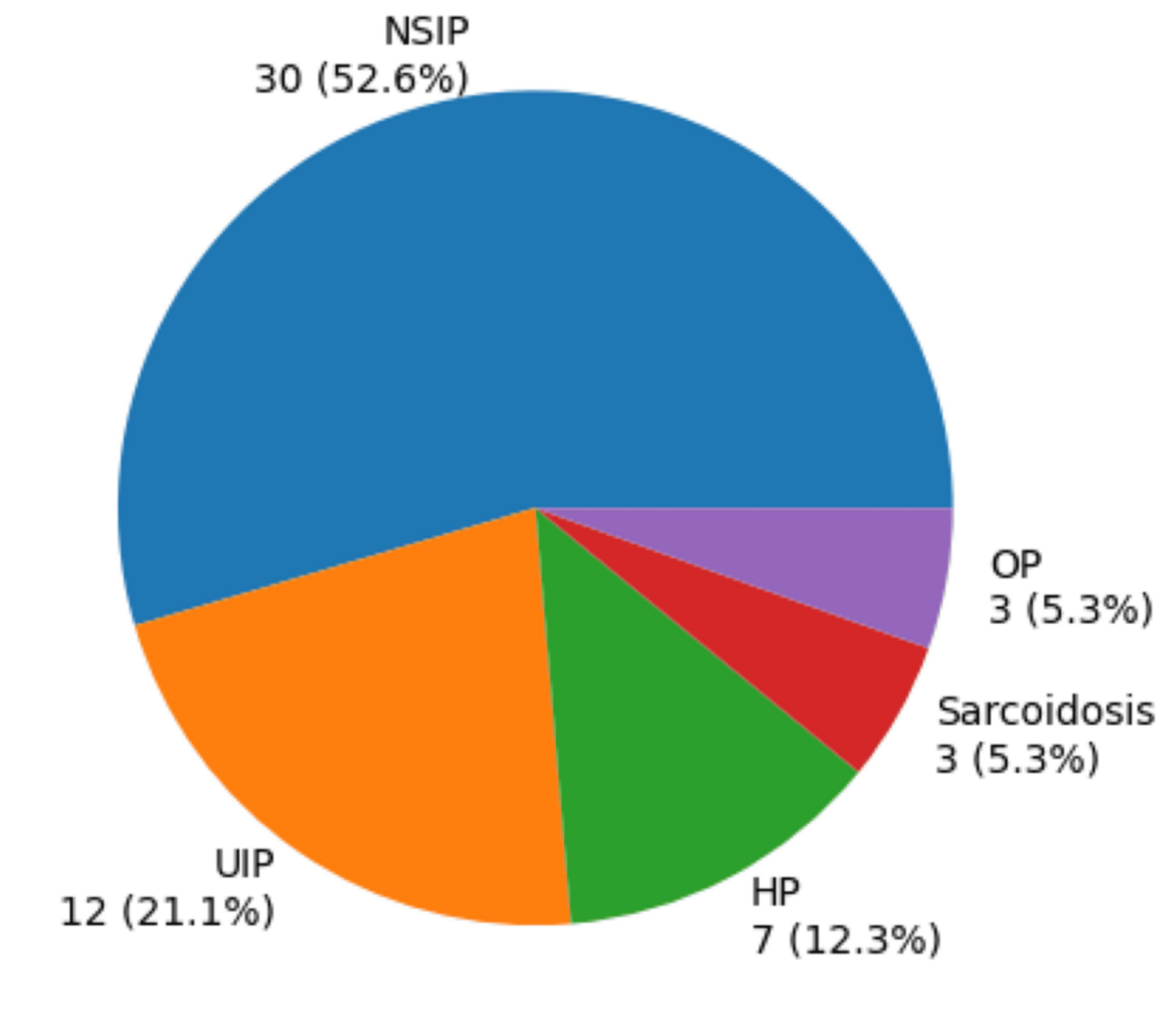
Distribution of ILD pattern. NSIP: nonspecific interstitial pneumonitis; UIP: usual interstitial pneumonitis; HP: hypersensitivity pneumonitis; OP: organizing pneumonia.

Figure 3 illustrates the distribution of the predominant HRCT patterns among the 57 patients with fibrosing ILDs. NSIP was the most common pattern, observed in 30 (52.6%). Usual interstitial pneumonia (UIP) was the second most frequent, seen in 12 (21.1%) patients. Hypersensitivity pneumonitis (HP) accounted for seven (12.3%), followed by sarcoidosis and organizing pneumonia (OP) in three each (5.3%).

**Figure 4:**
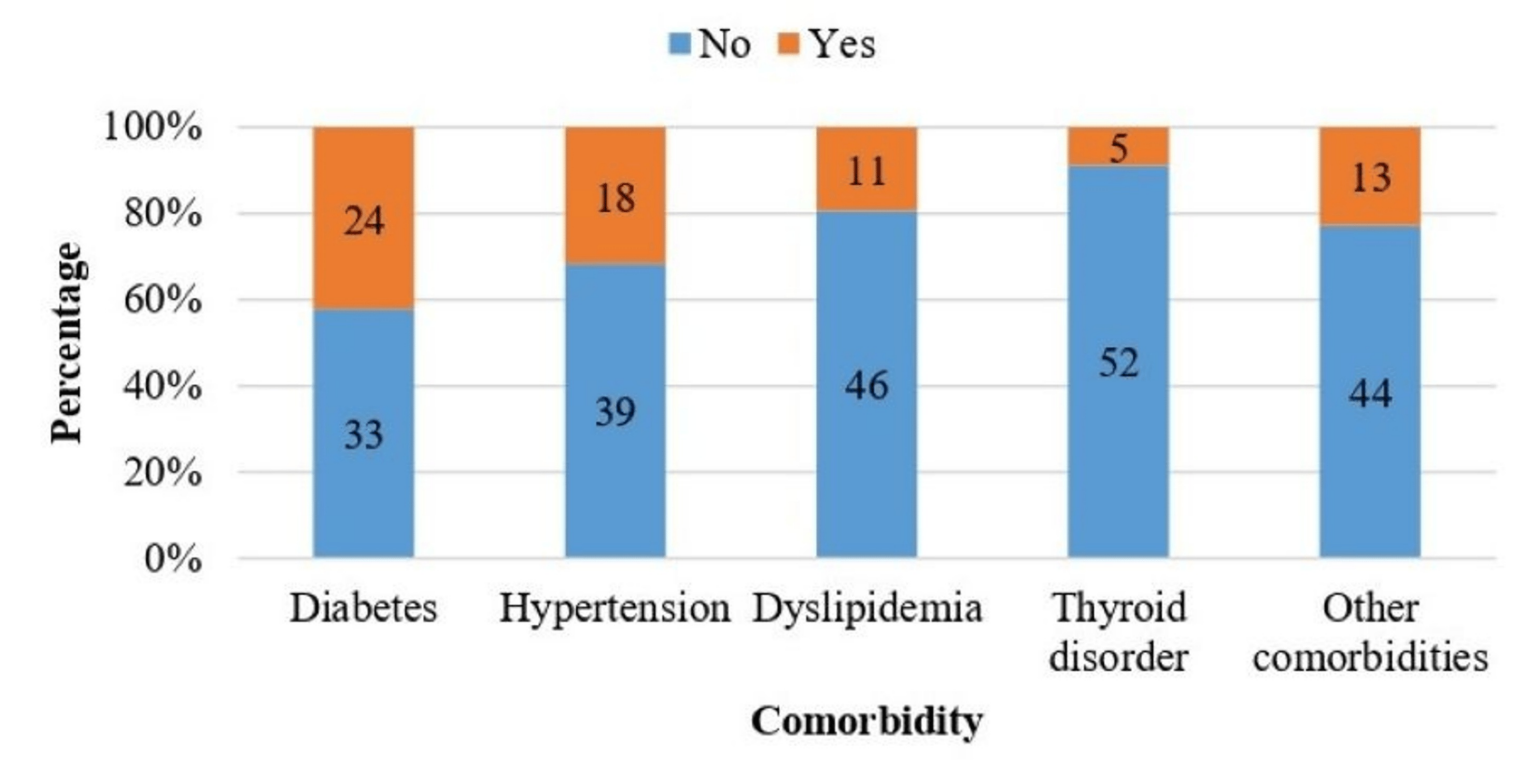
Distribution according to comorbidity status.

Figure 4 depicts the distribution of comorbidities in the study subjects. Diabetes mellitus was the most common comorbidity, present in 24 (42.1%), followed by hypertension in 18 (31.6%), dyslipidemia in 11 (19%), thyroid disorder in five (8.8%), and other comorbidities in 13 (22.8%).

**Figure 5:**
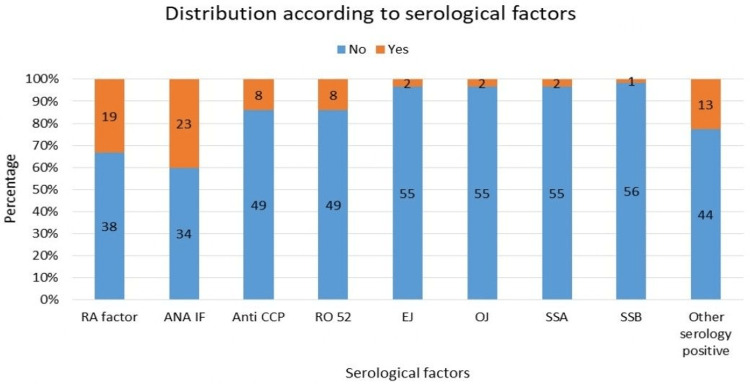
Distribution according to serological factors. RA: rheumatoid arthritis; ANA-IF: antinuclear antibody immunofluorescence; Anti-CCP: anti-cyclic citrullinated peptide antibody; Ro52: autoantibody directed against the Ro52 protein, part of the Ro-SSA family; EJ: anti-EJ is an anti-aminoacyl tRNA synthetase antibody; OJ: anti-OJ autoantibody directed against isoleucyl-tRNA synthetase; SSA: Sjogren's syndrome A antibody; SSB: Sjogren's syndrome B antibody.

Figure 5 depicts the distribution of patients according to serological status. Serological testing revealed positivity for rheumatoid factor (RF) in 19 (33.3%), antinuclear antibody (ANA) by immunofluorescence in 23 (40.4%), anti-cyclic citrullinated peptide (anti-CCP) in eight (14.0%), and anti-Ro-52 in eight (14.0%). Additionally, myositis-associated autoantibodies (anti-EJ, anti-OJ) and Sjogren's-related markers (anti-SSA, anti-SSB) were positive in a minority of cases.

Table 5 presents the mean values (with standard deviations) of key study variables at baseline and at the six-month follow-up.

**Table 5:** Mean values (with standard deviation) of the study variables at baseline and at six-month follow-up. FVC: forced vital capacity; 6MWT: six-minute walk test; GG: ground glass; LTA: lung texture analysis; %: percentage.

Parameter	Baseline (Mean ± SD)	6 months (Mean ± SD)
Dyspnea	1.23 (0.46)	2.25 (0.83)
FVC	64.77 (19.9)	63.30 (21.6)
6MWT - Distance	244.21 (74.23)	228.85 (83.64)
6MWT - Desaturation	3.28 (3.97)	3.81 (3.56)
Warrick alveolitis index (GG)	2.60 (0.98)	2.44 (0.95)
LTA ground glass score	33.82 (27.33)	28.88 (24.98)
Warrick fibrosis score	9.35 (5.46)	9.79 (5.48)
LTA fibrosis score	9.72 (8.73)	10.09 (8.73)
Warrick total	11.95 (5.28)	12.23 (5.53)
LTA normal %	54.46 (27.11)	61.04 (26.41)

Longitudinal changes in clinical, functional, and radiological parameters

The mean dyspnea score increased from 1.23 ± 0.46 at baseline to 2.25 ± 0.83 at six months. The mean forced vital capacity (FVC % predicted) declined from 64.8 ± 19.9% at baseline to 63.3 ± 21.6% at six months. The mean six-minute walk distance (6MWD) decreased from 244.2 ± 74.2 meters to 228.9 ± 83.6 meters at six months. The mean oxygen desaturation during the 6MWT increased from 3.28 ± 3.97% to 3.81 ± 3.56%.

Regarding radiological parameters, the LTA fibrosis score (sum of LTA-derived honeycombing and reticulation scores) increased from 9.72 ± 8.73 at baseline to 10.09 ± 8.73 at six months. Similarly, the Warrick fibrosis score increased from 9.35 ± 5.46 to 9.79 ± 5.48. The mean LTA % normal lung was 54.46 ± 27.11% at baseline and 61.04 ± 26.41% at six months.

Correlation of CALIPER with HRCT scoring

**Figure 6:**
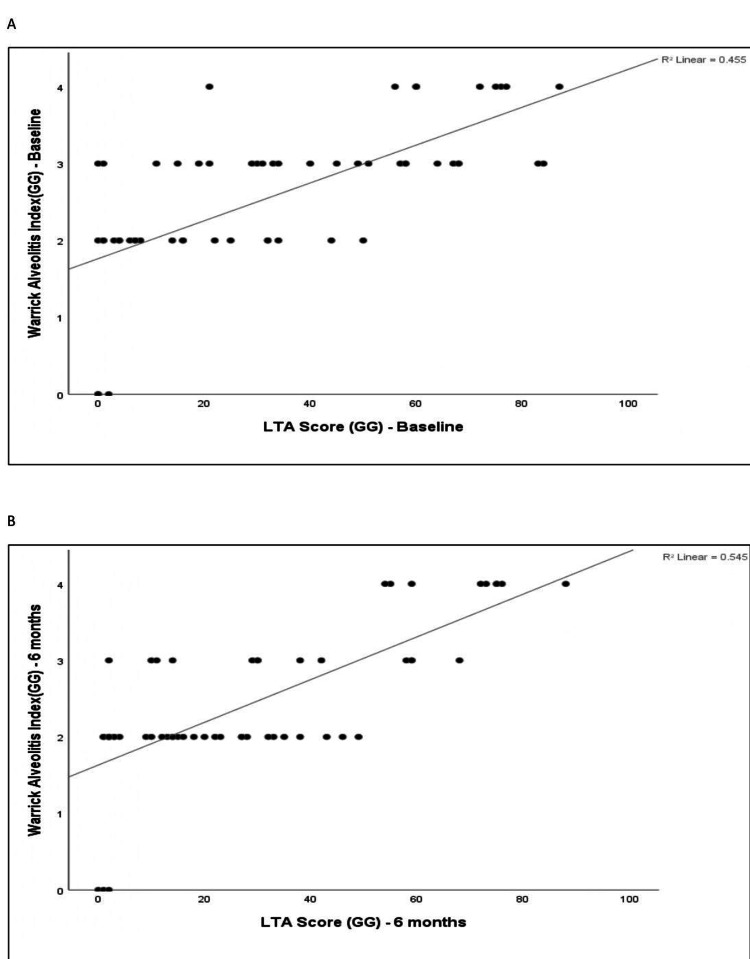
Scatter plot of the Warrick alveolitis index (GG) vs. LTA score (GG) at baseline and at six-month follow-up. (A) Scatter plot of the Warrick alveolitis index (GG) vs. LTA score (GG) at baseline. (B) Scatter plot of Warrick alveolitis index (GG) vs. LTA score (GG) at six-month follow-up. GG: ground glass; LTA: lung texture analysis.

The correlation between quantitative LTA metrics (ground-glass extent) and the Warrick alveolitis indices was assessed at baseline and at six-month follow-up. The baseline LTA-derived ground-glass (GG) percentage showed a moderate positive correlation with the baseline Warrick alveolitis index (r = 0.675, p < 0.001). At six months, LTA-derived ground-glass extent showed a high positive correlation with the Warrick alveolitis index (r = 0.700, p < 0.001). The relationships are illustrated by scatter plots in Figure 6.

**Figure 7:**
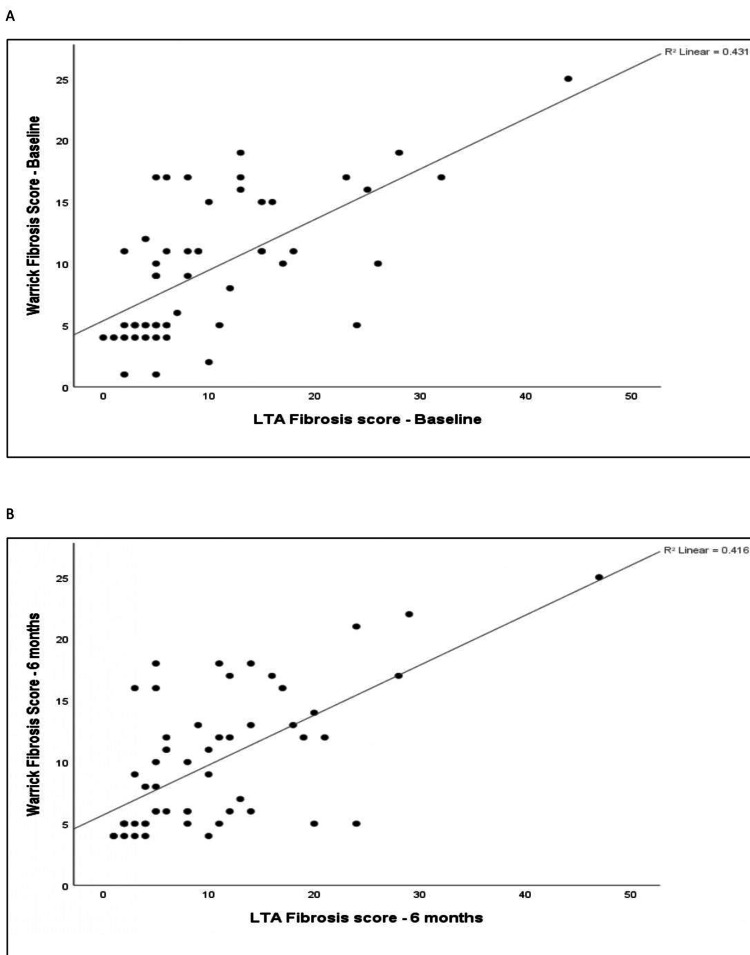
Scatter plot of the Warrick fibrosis score vs. the LTA fibrosis score at baseline and at six-month follow-up. (A) Scatter plot of the Warrick fibrosis score vs. the LTA fibrosis score at baseline. (B) Scatter plot of the Warrick fibrosis score vs. the LTA fibrosis score at six-month follow-up. LTA: lung texture analysis.

The correlation between quantitative LTA metrics (fibrosis extent) and the Warrick fibrosis index was assessed at baseline and six-month follow-up. The baseline LTA fibrosis score demonstrated a moderate positive correlation with the baseline Warrick fibrosis score (r = 0.656, p < 0.001). Similarly, the LTA fibrosis score at six months demonstrated a moderate positive correlation with the Warrick fibrosis score at six months (r = 0.631, p < 0.001). The relationships are illustrated by scatter plots in Figure 7.

Correlation of CALIPER with functional parameters

**Figure 8:**
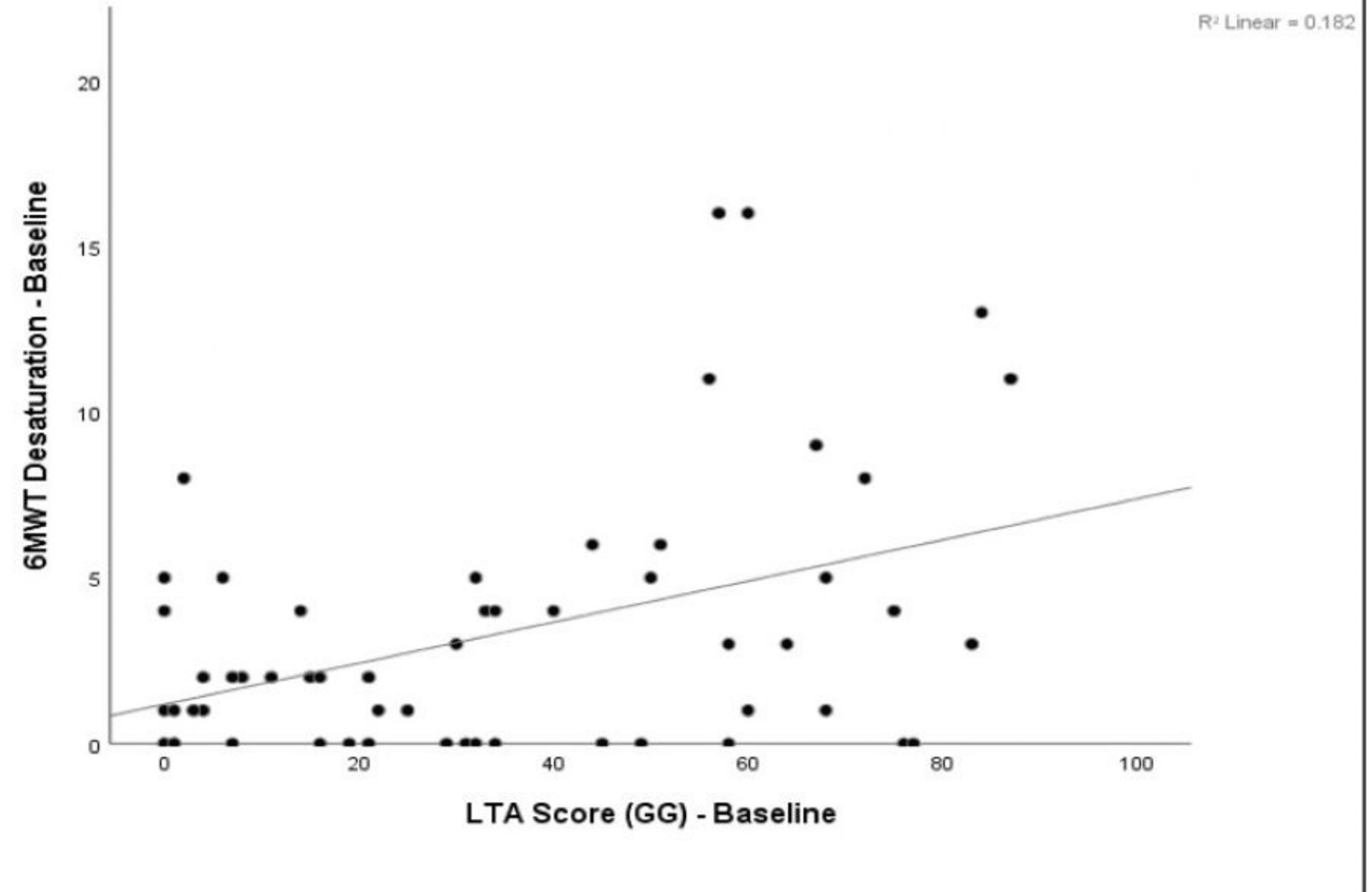
Scatter plot of 6MWT desaturation at baseline vs. LTA score (GG) at baseline. 6MWT: six-minute walk test; GG: ground glass; LTA: lung texture analysis.

The correlation between baseline LTA-derived ground-glass extent and functional parameters at baseline was studied. Baseline LTA-derived ground-glass (GG) extent showed a negligible negative correlation with forced vital capacity (FVC % predicted) (r = −0.241, p = 0.071), which was not statistically significant. Similarly, there was a negligible negative correlation between baseline LTA GG extent and baseline 6MWD (r = −0.010, p = 0.943), also not statistically significant. In contrast, baseline LTA GG extent demonstrated a low positive correlation with baseline 6MWT desaturation (r = 0.427, p = 0.001), which was statistically significant. Figure 8 displays a scatter plot of baseline 6MWT desaturation (%) versus baseline LTA-derived ground-glass extent.

**Figure 9:**
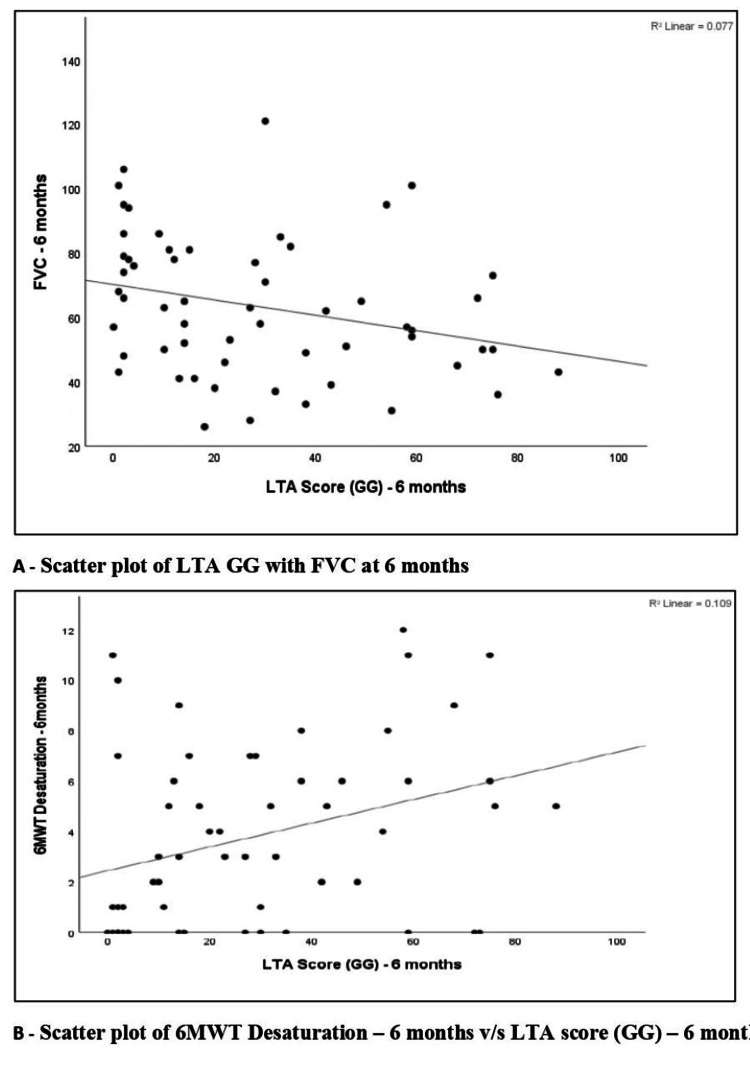
Scatter plot of LTA GG with FVC at six months and scatter plot of 6MWT desaturation at six months vs. LTA score (GG) at six months. (A) Scatter plot of FVC at six months vs. LTA score (GG) at six months. (B) Scatter plot of 6MWT desaturation at six months vs. LTA score (GG) at six months. FVC: forced vital capacity; 6MWT: six-minute walk test; GG: ground glass; LTA: lung texture analysis.

Spearman correlation between LTA-derived ground-glass extent at six months and functional parameters at six months was assessed. Baseline LTA-derived ground-glass extent at six months showed a low negative correlation with FVC % predicted at six months (r = −0.320, p = 0.015). It showed a negligible negative correlation with 6MWD at six months (r = −0.040, p = 0.766), which was not statistically significant. In contrast, LTA-derived ground-glass extent at six months demonstrated a low positive correlation with 6MWT desaturation at six months (r = 0.366, p = 0.011), which was statistically significant. The relationships are illustrated by scatter plots in Figure 9.

**Figure 10:**
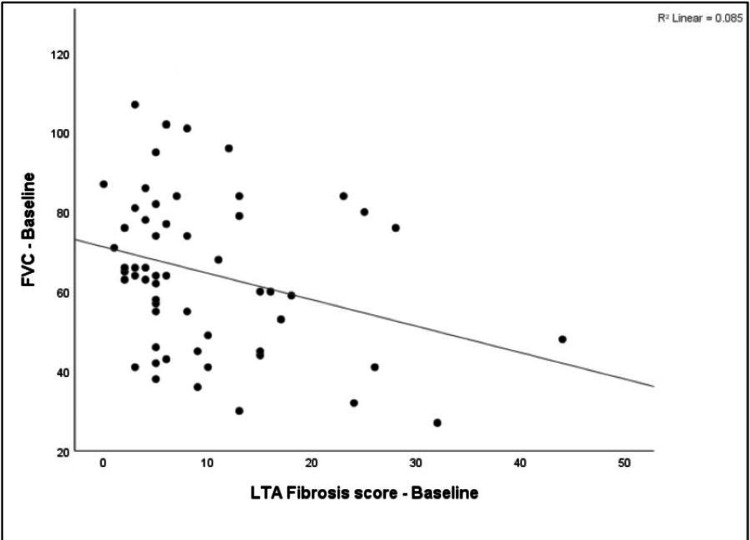
Scatter plot of FVC at baseline vs. LTA fibrosis score at baseline. FVC: forced vital capacity; LTA: lung texture analysis.

Spearman correlation between baseline LTA fibrosis score and functional parameters at baseline was evaluated. The baseline LTA fibrosis score showed a negligible negative correlation with baseline FVC % predicted (r = −0.292, p = 0.028). It showed a negligible negative correlation with baseline 6MWD (r = −0.130, p = 0.333), which was not statistically significant. Additionally, there was a negligible positive correlation with baseline 6MWT desaturation (r = 0.173, p = 0.199), which was not statistically significant. Figure 10 illustrates the scatter plot of the same.

**Figure 11:**
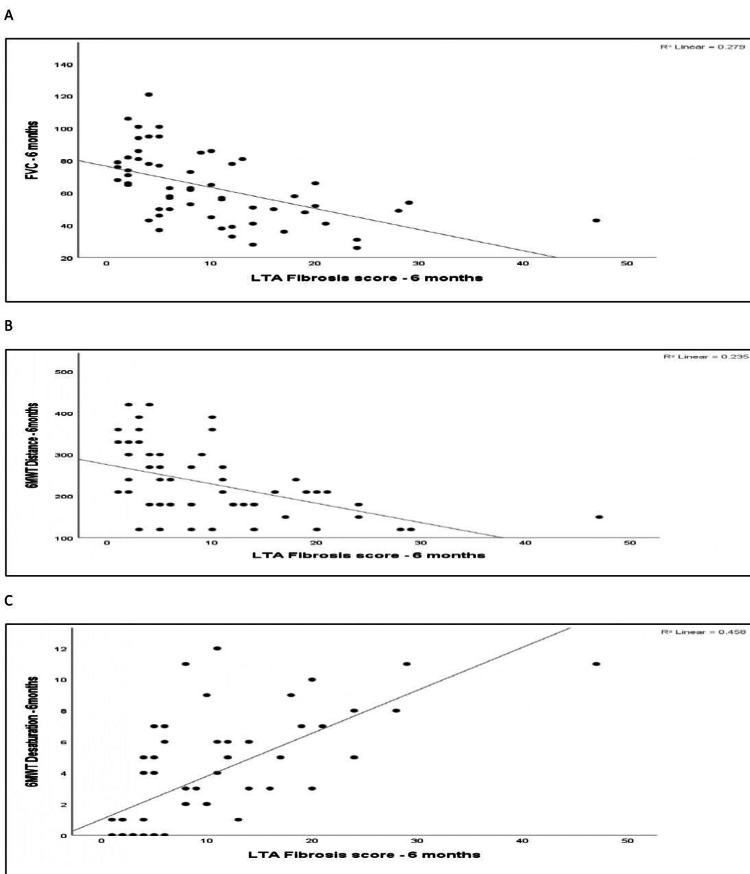
Scatter plot of FVC at six months vs. LTA fibrosis at six months, scatter plot of 6MWT distance at six months vs. LTA fibrosis score at six months, and scatter plot of 6MWT desaturation at six months vs. LTA fibrosis score at six months. (A) Scatter plot of FVC at six months vs. LTA fibrosis at six months. (B) Scatter plot of 6MWT distance at six months vs. LTA fibrosis score at six months. (C) Scatter plot of 6MWT desaturation at six months vs. LTA fibrosis score at six months. FVC: forced vital capacity; 6MWT: six-minute walk test; LTA: lung texture analysis.

Spearman correlation between the LTA fibrosis score at six months and functional parameters at six months was analyzed. The LTA fibrosis score at six months showed a moderate negative correlation with FVC % predicted at six months (r = −0.620, p < 0.001). It showed a moderate negative correlation with 6MWD at six months (r = −0.551, p < 0.001). In contrast, the LTA fibrosis score at six months demonstrated a strong positive correlation with 6MWT desaturation at six months (r = 0.731, p < 0.001). Figure 11 shows the scatter plots for the same.

**Figure 12:**
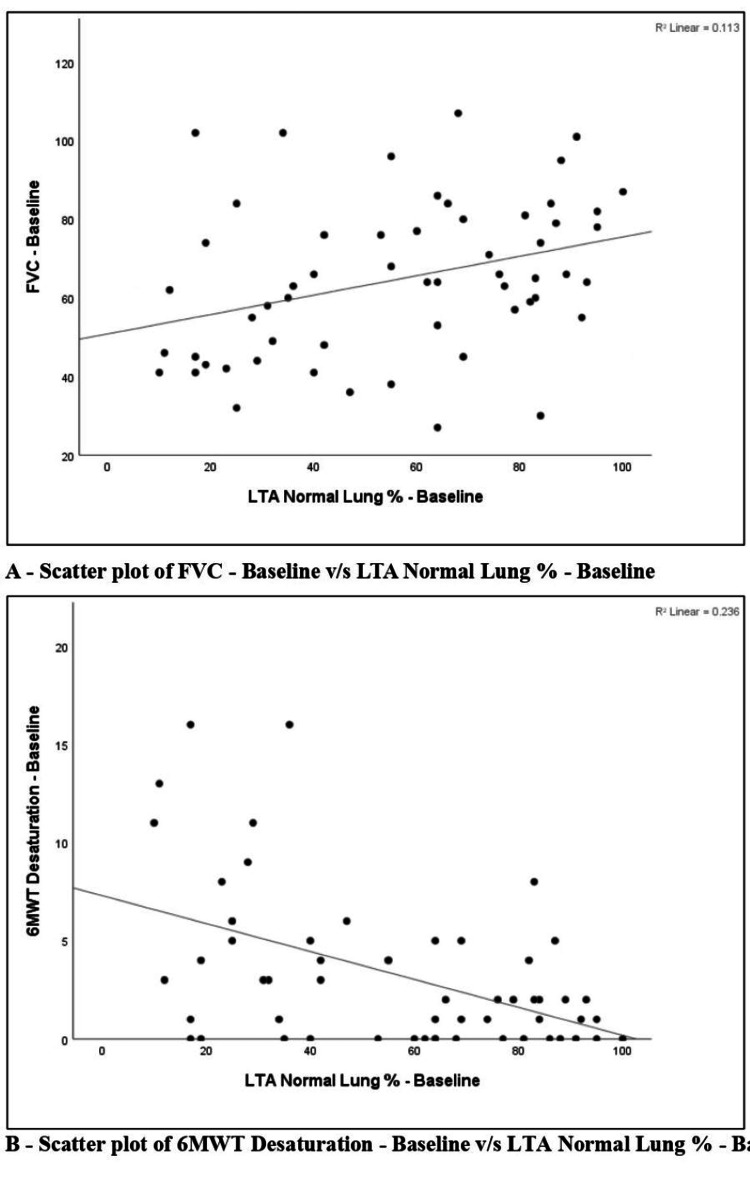
Scatter plot of FVC at baseline vs. LTA normal lung % at baseline and scatter plot of 6MWT desaturation at baseline vs. LTA normal lung % at baseline. (A) Scatter plot of FVC at baseline vs. LTA normal lung % at baseline. (B) Scatter plot of 6MWT desaturation at baseline vs. LTA normal lung % at baseline. FVC: forced vital capacity; 6MWT: six-minute walk test; LTA: lung texture analysis.

Spearman correlation between baseline LTA normal lung percentage and functional parameters was examined. Baseline LTA normal lung percentage (calculated as 100 minus the sum of LTA fibrosis and ground-glass scores) showed a low positive correlation with baseline FVC % predicted (r = 0.336, p = 0.010). It showed a negligible positive correlation with baseline 6MWD (r = 0.052, p = 0.702), which was not statistically significant. In contrast, it demonstrated a moderate negative correlation with baseline 6MWT desaturation (r = −0.486, p < 0.001), which was statistically significant. Figure 12 depicts the scatter plots for the same.

**Figure 13:**
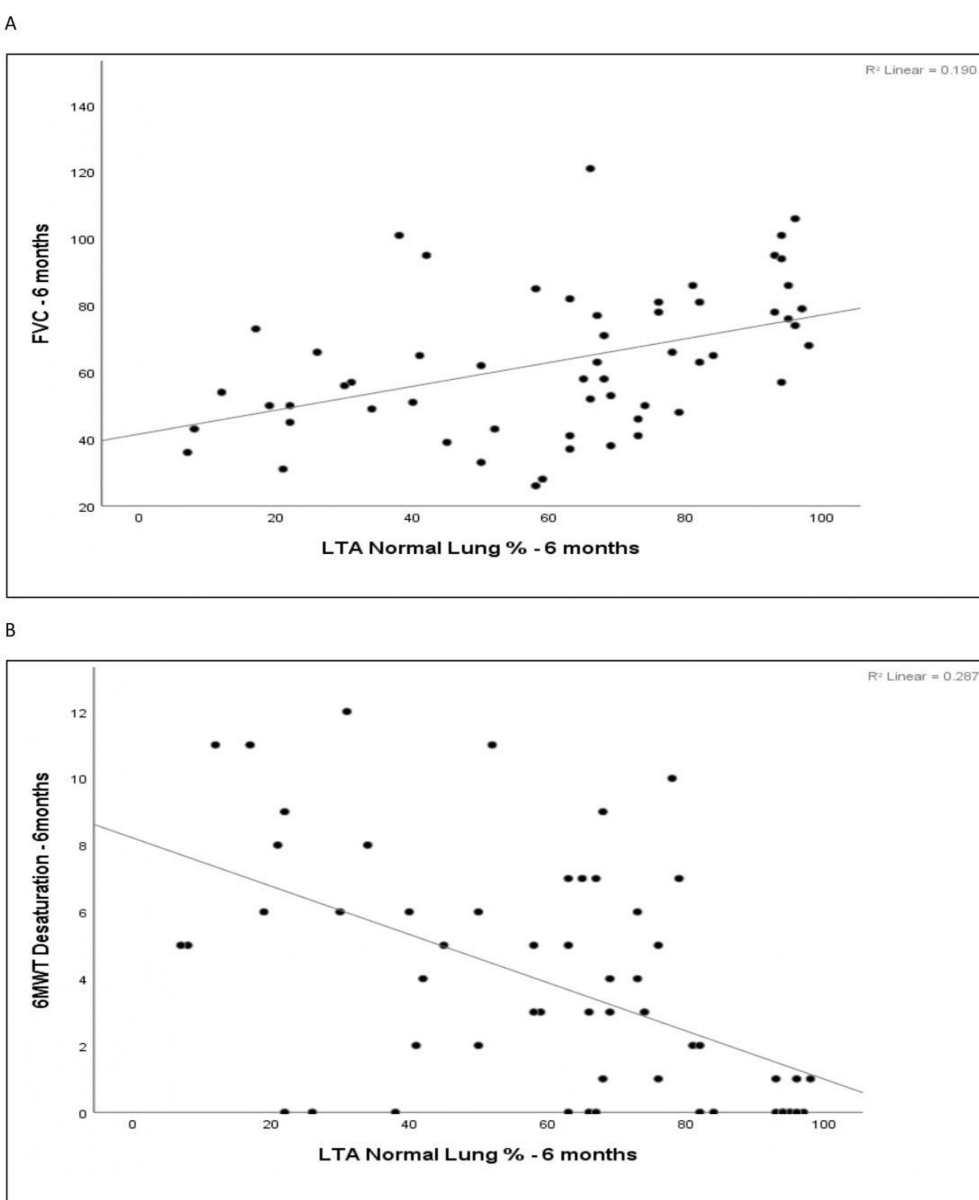
Scatter plot of FVC at six months vs. LTA normal lung % at six months, and scatter plot of 6MWT desaturation at six months vs. LTA normal lung % at six months. (A) Scatter plot of FVC at six months vs. LTA normal lung % at six months. (B) Scatter plot of 6MWT desaturation at six months vs. LTA normal lung % at six months. FVC: forced vital capacity; 6MWT: six-minute walk test; LTA: lung texture analysis.

Spearman correlation between LTA normal lung percentage at six months and functional parameters at six months was investigated. LTA normal lung percentage at six months showed a moderate positive correlation with FVC % predicted at six months (r = 0.480, p < 0.001). It showed a negligible positive correlation with 6MWD at six months (r = 0.213, p = 0.112), which was not statistically significant. In contrast, it demonstrated a moderate negative correlation with 6MWT desaturation at six months (r = −0.537, p < 0.001), which was statistically significant. Figure 13 depicts the scatter plots.

**Figure 14:**
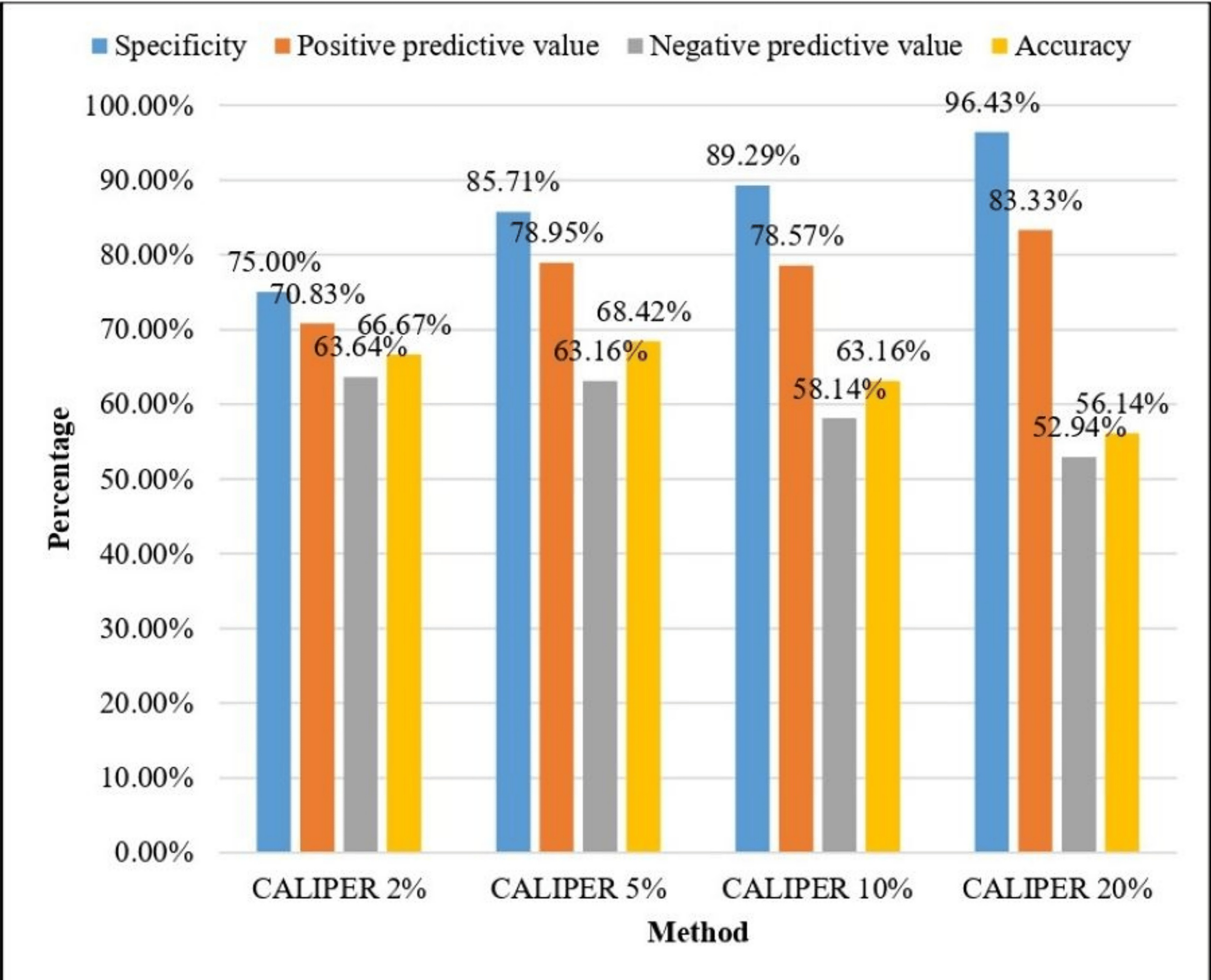
Diagnostic characteristics of various CALIPER cut-offs. CALIPER: Computer-Aided Lung Informatics for Pathology Evaluation and Rating.

The diagnostic performance of different CALIPER thresholds (2%, 5%, 10%, and 20% absolute change in normal lung percentage) for identifying PPF is shown in Table 6 and Figure 14.

**Table 6:** Comparison of diagnostic characteristics of various CALIPER cut-offs with progressive pulmonary fibrosis (PPF). CALIPER: Computer-Aided Lung Informatics for Pathology Evaluation and Rating.

Method	True positive	False positive	False negative	True negative	Sensitivity	Specificity	Positive predictive value	Negative predictive value	Accuracy
CALIPER 2%	17	7	12	21	58.62%	75.00%	70.83%	63.64%	66.67%
CALIPER 5%	15	4	14	24	51.72%	85.71%	78.95%	63.16%	68.42%
CALIPER 10%	11	3	18	25	37.93%	89.29%	78.57%	58.14%	63.16%
CALIPER 20%	5	1	24	27	17.24%	96.43%	83.33%	52.94%	56.14%

CALIPER 2% demonstrated the highest sensitivity (58.62%), indicating better ability to detect true positives, with a moderate specificity (75.00%) and overall accuracy of 66.67%. As the threshold increased to 5% and 10%, sensitivity progressively decreased (51.72% and 37.93%, respectively), while specificity increased (85.71% and 89.29%). CALIPER 20% showed the highest specificity (96.43%) and PPV (83.33%), but very low sensitivity (17.24%), meaning it missed a large number of true positive cases.

The 5% CALIPER change threshold demonstrated an acceptable balance between sensitivity and specificity (51.72% and 85.71%, respectively), with comparatively high PPV (78.95%), moderate NPV (63.16%), and the highest overall accuracy (68.42%) among the thresholds tested. The 10% threshold demonstrated moderate specificity (89.29%) but reduced sensitivity (37.93%) and lower accuracy (63.16%).

**Figure 15:**
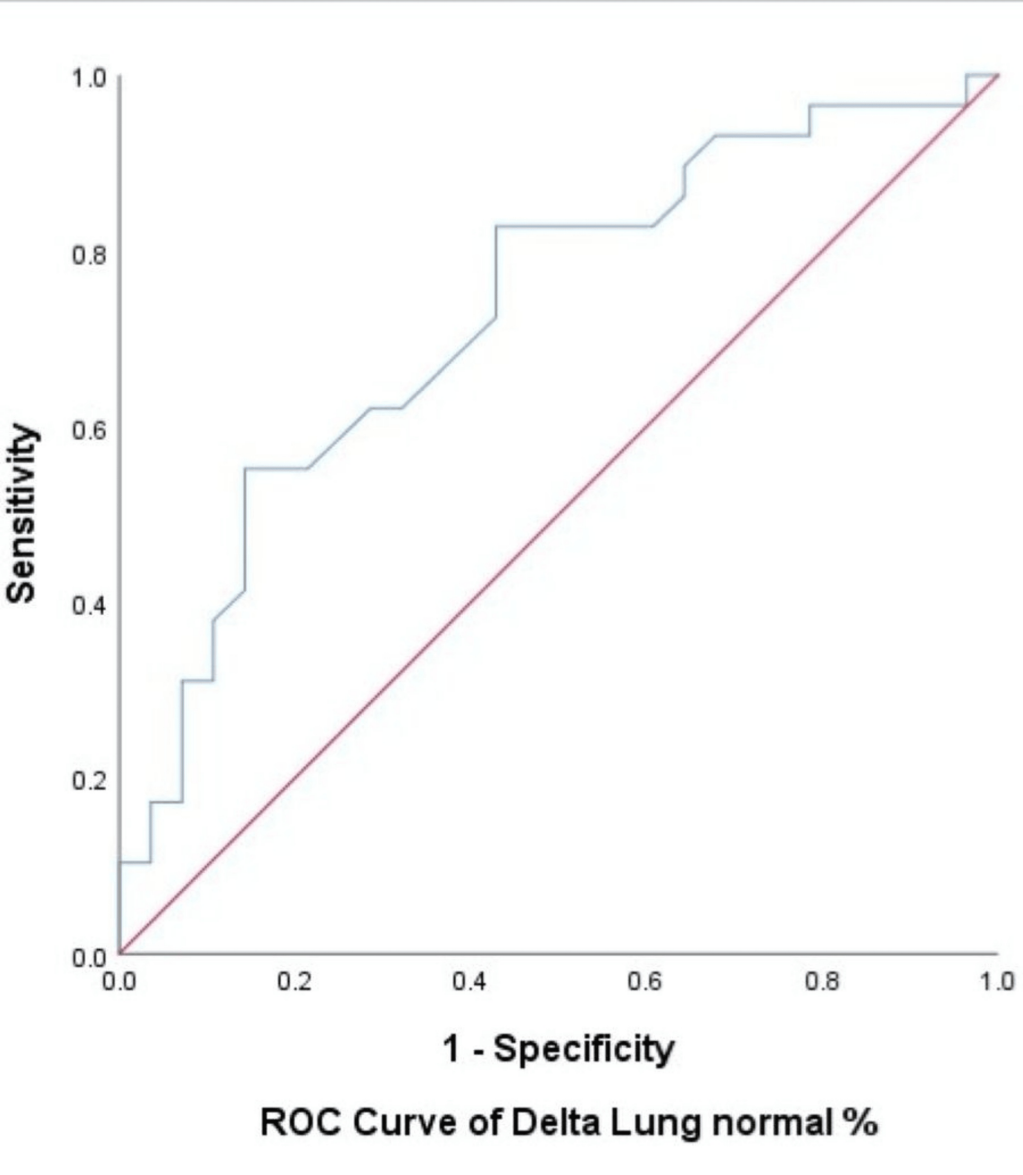
Receiver operating characteristic (ROC) curve for delta lung percentage (change in normal lung %) in predicting progressive pulmonary fibrosis (PPF).

ROC analysis was performed to assess the ability of the delta lung percentage to predict PPF. The ROC curve is shown in Figure 15.

Table 7 illustrates the diagnostic performance of the 5% cut-off for delta lung percentage in predicting PPF.

**Table 7:** Diagnostic performance of the 5% cut-off for delta lung percentage in predicting progressive pulmonary fibrosis (PPF).

Cut-off	Sensitivity	Specificity
5%	51.72%	85.71%

Table 8 shows the AUC for the delta lung percentage in predicting PPF.

**Table 8:** Area under the curve for delta lung % in predicting progressive pulmonary fibrosis (PPF).

Area	Std. error	P-value	95% CI	
			Lower bound	Upper bound
0.729	0.067	0.003	0.598	0.861

The AUC was 0.729 with a standard error of 0.067 and a 95% CI ranging from 0.598 to 0.861. Since the AUC is greater than 0.5 and the confidence interval does not include 0.5, the test demonstrates acceptable discriminatory ability to differentiate between positive and negative outcome groups. Furthermore, the p-value associated with the ROC curve was 0.003, which is statistically significant (p < 0.05). Therefore, the variable delta lung percentage significantly predicts the outcome when compared to PPF.

## Discussion

In a study by Ryerson et al. [17], 132 patients were included. Among the study population, 52 (40%) were males, and 80 (60%) were females. The mean age of the study population was 68 years. In another study by Occhipinti et al. [18], 35 patients were included. The mean age of the study population was 51.8 years. Out of these, eight (22%) were males, and 27 (77%) were females. In our study, the mean age was 57.8 ± 11.12 years. It was comparable to the mean age mentioned in the Indian ILD Registry by Singh et al. (55.4 ± 12.8) [3]. The female predominance in our study population may be due to the inclusion of a greater number of CTD-related ILD patients. In a study by Kim et al. [19] of rheumatoid arthritis-associated ILD and systemic sclerosis patients, a female predominance was observed (68%), which is comparable to this study, with a female predominance of 38 (66%).

In a study of 803 subjects by Dhooria et al., the most common ILD was sarcoidosis, accounting for 339 (42.2%) of cases, followed by IPF, with 170 cases (21.2%) [4]. Another study by Dhooria et al., analyzing 3,089 subjects to estimate the incidence, prevalence, and national burden of ILDs in India, found sarcoidosis to be the most frequent ILD (1152, 37.3%), with CTD-related ILD being the second most common (596, 19.3%) [20]. In our study, CTD-related ILD was the most prevalent, comprising 24 (42.1%) of cases, followed by idiopathic NSIP at 15 (26.3%). The predominance of CTD-related ILD in the present study was comparable to the above-mentioned studies.

In a study by Canofari et al. [21], the most common serological marker identified was ANA by immunofluorescence (ANA IF), followed by anti-Ro as the second most common marker. In our study, the most common serological marker was ANA IF, which was positive in 23 (40.35%) patients, followed by rheumatoid arthritis factor, which was positive in 19 (33.33%) patients.

Upon considering the longitudinal changes in respiratory symptoms and functional parameters over six months, there was an increase in LTA-derived fibrosis, along with a decline in 6MWT desaturation, distance, and FVC, with worsening dyspnea grade.

Correlation analysis demonstrated a significant association between quantitative CALIPER-derived parameters and semi-quantitative Warrick scoring. The correlation analysis showed that LTA ground glassing (GG) driven by CALIPER at baseline had a moderate positive correlation with the Warrick alveolitis index at baseline (r = 0.675, p < 0.001), indicating a statistically significant association. Whereas LTA GG at six months had a high positive correlation with the Warrick alveolitis index at six months (r = 0.700, p < 0.001), indicating a statistically significant and strong association between the two measures. The mean Warrick alveolitis index was 2.60 at baseline and 2.44 at six months, while the mean LTA ground glassing decreased from 33.82% to 28.88%.

The correlation analysis demonstrated that the LTA fibrosis score at baseline and at six months had a moderate positive correlation with the Warrick fibrosis score at baseline (r = 0.656, p < 0.001) and six months (r = 0.631, p < 0.001), respectively. Both of these associations were statistically significant. This is comparable to a study by Occhipinti et al. [18], in which quantitative analysis had a weak-to-good concordance with semi-quantitative analysis, with an intraclass correlation coefficient of 0.275 for reticular and 0.667 for ground-glassing.

In a study done by Salisbury et al., the authors found that there is a moderate agreement between visual and quantitative measures with ground glass reticulation (Pearson’s correlation r = 0.60, p < 0.0001) [22]. In IPF, visually assessed disease extent on CT scans is a powerful independent predictor of mortality. While the quantitative CT scans taken serially will be useful for assessing the disease progression in ILD, as shown by a study done by Best et al. [13]. These findings support the validity of CALIPER-derived quantitative metrics as reliable surrogates for visual HRCT scoring systems.

The LTA fibrosis score at six months showed a moderate negative correlation with FVC, with a Spearman correlation coefficient (r = −0.620, p < 0.001), indicating that as LTA-derived fibrosis increases, FVC declines. The mean LTA fibrosis score was 9.72% at baseline, and increased to 10.09% at six months. The adaptive multiple features method (AMFM) is an automated HRCT analysis, and the AMFM-measured ground glass reticular trajectory was weakly negatively correlated with post-baseline FVC trajectory [23].

The LTA fibrosis score at six months showed a high positive correlation with 6MWT desaturation (r = 0.731, p < 0.001), indicating that as LTA-derived fibrosis worsens, 6MWT desaturation increases. The LTA fibrosis score at six months showed moderate negative correlation with 6MWT distance (r = −0.551, p < 0.001), which means that as the fibrosis driven by LTA rises, the 6MWT distance falls. These findings indicate that increasing structural fibrosis is strongly associated with worsening exercise capacity and gas exchange impairment. Du Bois et al. described that the 6MWT desaturation is a good predictor of mortality in fibrotic ILDs [24]. Likewise, Ley et al. demonstrated that spirometry alone is not a good predictor, whereas the physiological and imaging indices should be combined for evaluation of ILDs [23].

The LTA ground glass at baseline was showing low or negligible correlation with functional indices like FVC, 6MWT distance, and a low positive correlation with 6MWT desaturation (r = 0.427, p = 0.001). This may be due to many factors. Ground glassing in CT may be due to infections like viral or atypical pneumonia, pulmonary edema, eosinophilic pneumonia, organizing pneumonia, and sometimes, when there is a significant motion artifact in the CT image, the CALIPER may misinterpret it as ground glass abnormality.

LTA normal lung percentage at six months demonstrated a moderate negative correlation with 6MWT desaturation at six months (r = −0.537, p < 0.001), indicating a statistically significant association, where a higher normal lung percentage was associated with lower desaturation. Jacob et al. demonstrated that preserved normal lung volume may be considered as an independent predictor of survival in fibrosing ILD [14].

Four different cut-offs (2%, 5%, 10%, and 20%) were explored. Here, we found that CALIPER 2% and 5% change in normal lung volume % had the greatest sensitivities, so these cut-offs are good to capture true progressors as compared to the higher cut-offs. The CALIPER 5% change in normal lung volume % showed a diagnostic accuracy of 68.42% and maintained an acceptable balance between sensitivity (51.72%) and specificity (85.71%). In clinical practice, this equilibrium is especially crucial because excessively sensitive thresholds (for example, cut-offs less than 2%) could result in overdetection of progression and unnecessary treatment escalation, but overly precise thresholds might overlook the onset of the disease.

The CALIPER 20% threshold had the highest specificity (96.43%), but its low sensitivity (17.24%) significantly reduces its usefulness for early progression detection. When confirming severe progression, high specificity and low sensitivity might be acceptable, but not for routine longitudinal monitoring, where early detection is crucial. The 5% threshold appears to offer an optimal balance by capturing early structural changes while minimizing false-positive results.

Progressive pulmonary fibrosis is known to develop radiologically before functional impairment occurs, and early quantitative changes may precede measurable declines in FVC or DLCO. Lower thresholds, such as 2%, increase sensitivity but will have a higher false-positive rate (possibly detecting measurement variability and not biological change), while thresholds above 10% will have decreased sensitivity and miss early progression events. The 5% threshold optimizes the correct classification of both progressors and non-progressors and is best suited for clinical decision-making.

Research analyzing quantitative CT parameters has shown that, regardless of spirometry, incremental increases in fibrosis volume above 4-6% are linked to worse outcomes and higher mortality. This further supports the 5% threshold’s clinical relevance. Image quality, reconstruction parameters, and respiratory phase all contribute to the inherent variability of quantitative CT algorithms, such as CALIPER. Therefore, thresholds below 2-3% might represent “noise” instead of meaningful biological advancement. The 5% threshold captures early disease changes while offering a margin above this inherent variability.

CALIPER thresholds enable clinicians to reduce misdiagnosis, identify early structural progression prior to measurable functional decline, and identify patients for closer follow-up or early initiation of antifibrotic therapy.

Limitations

The present study has certain limitations. The relatively short duration of follow-up may have limited the ability to identify all cases of progressive ILD. In addition, some patients may have received prior treatment before presentation to our center, which could have led to non-identification of disease progression.

## Conclusions

Quantitative LTA using CALIPER demonstrates a moderate-to-high correlation with semi-quantitative Warrick HRCT scoring, along with a significant association with functional indices, such as the modified Medical Research Council dyspnea grading, FVC, and 6MWT parameters. As an objective and reproducible tool, CALIPER offers a reliable method for assessing disease burden in fibrosing ILDs, with a 5% change in normal lung volume emerging as a potential optimal threshold for clinically meaningful change.

Overall, CALIPER may serve as a valuable adjunct in the routine monitoring of fibrosing ILDs, enabling earlier identification of patients at risk for PPF and facilitating timely clinical decision-making.
